# Characterization of biofilm formation by *Enterococcus faecalis* isolates derived from urinary tract infections in China

**DOI:** 10.1099/jmm.0.000647

**Published:** 2017-11-17

**Authors:** Jin-xin Zheng, Bing Bai, Zhi-wei Lin, Zhang-ya Pu, Wei-ming Yao, Zhong Chen, Duo-yun Li, Xiang-bin Deng, Qi-wen Deng, Zhi-jian Yu

**Affiliations:** Department of Infectious Diseases and Shenzhen Key Lab for Endogenous Infection, Shenzhen Nanshan Hospital, Shenzhen University, No 89, Taoyuan Road, Nanshan District, Shenzhen 518052, PR China

**Keywords:** *Enterococcus faecalis*, biofilm, urinary-tract infections, virulence genes, multilocus sequence typing

## Abstract

**Purpose:**

This study explored the prevalence and characteristics of *Enterococcus faecalis* biofilm formation by urinary tract infection (UTI) isolates in order to identify virulence factors associated with biofilm formation.

**Methodology:**

A total of 113 *E. faecalis* isolates were collected from UTI patients in Shenzhen, China. The isolates were subjected to multilocus sequence typing based on housekeeping genes. Biofilms were detected by crystal violet staining and the expression levels of the *E. faecalis* genes were detected by quantitative real-time PCR.

**Results/Key findings:**

The main sequence types (STs) were ST16 and ST179 with the ST16 isolates more likely to form strong biofilms than the ST179 isolates (*P=0.008*). Strong biofilm formation was more frequently detected in aggregation substance (*agg*)-positive (+) isolates than in negative (−) isolates (*P=0.033*). Biofilm formation was also more common in isolates containing enterococcal surface protein (*esp*), or cytolysin A (*cylA*)-positive (+) isolates than in isolates negative (−) for these virulence factors. Multivariate regression analysis indicated that *cylA* [odds ratio (OR), 7.143, *P*=*0.012*] was associated with weak biofilm formation, and that *agg* (OR, 4.471, *P=0.004*) was associated with strong biofilm formation. The expression of *cylA* was increased (8.75- to 23.05-fold) in weak biofilm, and the expression of *agg* was greatly elevated (11.99- to 439.10-fold) in strong biofilm isolates when compared to biofilm-negative isolates.

**Conclusion:**

ST16 classification was positively associated with strong biofilm formation in *E. faecalis* as was *agg*, while *cylA* was associated with weak biofilm formation.

## Introduction

Urinary tract infections (UTIs) are common in women and are one of the most frequent human bacterial infections [1]. With 150 million cases per year, UTIs are responsible for significant worldwide morbidity and loss of workplace productivity [2]. Although most UTIs (80 %–90 %) are caused by extra-intestinal *Escherichia coli* strains, recently *Enterococcus faecalis* strains have more frequently been isolated and have been reported to be causative agents in up to 20 % of all cases [3, 4]. *E. faecalis* UTIs are of particular concern in that they are intrinsically resistant to first-line antimicrobial agents, especially vancomycin [5]. Another concern is that *E. faecalis* strains can form biofilms that are difficult to eradicate with one report demonstrating that 100 % [6] and another that 70 % of urinary tract *E. faecalis* strains can form biofilms [7]. However, in China the prevalence and characteristics of *E. faecalis* UTI strains and their capacity to form biofilms is unknown.

Phylogenetic relationships of bacterial pathogens can be investigated by multilocus sequence typing (MLST), including *Staphylococcus* spp. and *Enterococcus* spp. [8]. The virulence and drug resistance of strains are distinctly different between various sequence types (STs) [9]. Biofilm formation, another important characteristic of bacteria, is also diverse between various ST isolates [10]. For example, *Staphylococcus epidermidis* ST27 was found to occur preferentially in hospitals and differed from community isolates in that hospital ST27 had the capacity to form biofilms [10]. ST17 and ST19 lineages of group B *Streptococcus* strains derived from invasive disease were significantly more likely to form weak biofilms than strains recovered from asymptomatic individuals that formed strong biofilms [11]. The relationship of various *E. faecalis* STs to biofilm formation has not been explored.

There are several virulence factors that are related to *E. faecalis* biofilm formation. The enterococcal surface protein (*esp*) has been found to adhere to and colonize abiotic surfaces, playing an important role in *E. faecalis* biofilm formation [12, 13]. *E. faecalis* gelatinase (*gelE*) is an extracellular metalloprotease, which can hydrolyse gelatin, collagen and haemoglobin, and is reported to be involved in bacterial adherence and biofilm formation [14, 15]. In a large collection of *E. faecalis* isolates no significant relationship among *esp, gelE* and biofilm formation was found [16–18]. Likewise, *E. faecalis* aggregating substance (*agg*) was found to promote biofilm formation by Chuang-Smith *et al*., but that report was not confirmed by other studies [19]. Thus, the association of virulence factors with *E. faecalis* biofilm formation is controversial and unresolved.

The aims of this study are to explore the prevalence and characteristics of UTI *E. faecalis* isolates, to assess their capacity to form biofilms, and to probe STs and virulence factors associated with biofilm formation. To our knowledge this is the first study to simultaneously assess biofilm formation, virulence genes, antimicrobial resistance and MLST for *E. faecalis* UTI isolates from China. As such, this study will provide a better understanding of *E. faecalis* and its capacity to form biofilms.

## Methods

### Bacterial isolates

A total of 113 *E. faecalis* isolates were obtained from the urine of UTI patients at Shenzhen Nanshan Hospital, Shenzhen University, China between 1 January 2011 and 30 June 2016. Bacterial isolates from patients were identified by the VITEK 2 system (Biomérieux, Marcy l’Etoile, France). *E. faecalis* ATCC29212 and OG1RF (ATCC47077) were used as reference strains and obtained from the American Type Culture Collection (ATCC).

### Antimicrobial susceptibility testing

Isolates' susceptibility to the clinically relevant antimicrobials; penicillin G, ampicillin, ciprofloxacin, teicoplanin, vancomycin, linezolid, high-level gentamicin, erythromycin and tetracycline was assessed with the VITEK 2 system (Biomérieux, Marcy l’Etoile, France). The MIC of ampicillin, vancomycin and linezolid were assessed by the broth macrodilution method according to the Clinical and Laboratory Standards Institute (CLSI) guidelines. The MIC breakpoints were determined based on the guidelines of CLSI for 2016.

### Isolation of DNA

The DNA from all isolates was extracted and purified using DNeasy Blood and Tissue Kits (QIAGEN China, Zhangjiang Hi-Tech Park Pudong, Shanghai, PR China) according to the manufacturer’s protocol for Gram-positive bacteria. The microbial DNA was eluted with 100 µl AE buffer (Qiagen) and stored at −20 °C.

### Detection of virulence genes by PCR

The extracted DNA served as a template for the amplification of virulence genes. All primer sequences and corresponding references are listed in Table S1 (available in the online version of this article) [20, 21]. PCR amplification was performed in a total volume of 50 µl, containing 2X PCR Master Mix (Tiangen Biotech (Beijing)), 0.5 mM of each of the primers and 1 µl of template DNA. The cycling conditions were as follows: 95 °C for 3 min, followed by 30 cycles of 95 °C for 30 s, 52 °C for 30 s and 72 °C for 60 s, with final extension at 72 °C for 10 min. Each set of PCR reactions included a no-template control and a positive control. The amplification products were analysed by gel electrophoresis using a 1.0 % agarose gel.

### *E. faecalis* MLST and clonal complex (CC) testing

MLST of *E. faecalis* isolates was performed according to the reference method [22]. Briefly, the seven housekeeping genes; *gdh*, *gyd*, *pstS*, *gki*, *aroE*, *xpt* and *yqiL* were PCR amplified and sequenced. All primer sequences and corresponding references are listed in Table S2. The PCR reaction system and cycling conditions were as described above, except for the primers. For each locus, a distinct allele number was assigned to each sequence, in accordance with the *E. faecalis* MLST database (http://efaecalis.mlst.net/). Allelic profile or STs were assigned in the order; *gdh*, *gyd*, *pstS*, *gki*, *aroE*, *xpt* and *yqiL* to a total of seven integers corresponding to the allele numbers at the seven loci. STs were assigned to isolates in such a way that the same ST names were consistent with the same isolates analysed. *E. faecalis* CCs were analysed by the eBURST v 3.0 program [23]. CCs were defined as groups of two or more independent isolates that shared identical alleles at six or more loci; each complex was named after the putative founder ST.

### Biofilm assay

Biofilm formation by *E. faecalis* isolates was done according to the reference method with modifications [24]. Briefly, the *E. faecalis* isolates were cultivated overnight in Tryptic Soy Broth (TSB) (OXOID; Basingstoke, Hampshire, UK) at 37 °C in ambient air for 10–12 h and shaken at 220 r min^−1^. Overnight cultures were diluted 1 : 100 in 200 µl of TSBG (TSB with 0.25 % glucose) (1.0–3.0×10^7^ c.f.u. ml^−1^) and inoculated into polystyrene microtitre plates (Costar3599, Corning). After 24 h of static incubation at 37 °C, the supernatant was discarded, and plates were washed thrice with deionized water to remove unattached cells, fixed with methanol for 30 min, stained with 1 % crystal violet (CV) for 30 min, and rinsed with distilled water. The optical density at 570 nm (OD_570_) was determined. Each assay was performed in triplicate at least three times.

### Quantitative real-time PCR

The expression levels of *esp, agg* and *cylA* genes of *E. faecalis* clinical isolates were detected by quantitative real-time (qRT)-PCR. The primers used for qRT-PCR are listed in Table S3. The RNA extraction was conducted using the reference method [25] with modification. Briefly, the *E. faecalis* isolates were cultured at 37 °C until the OD_600_ reached 0.6, the cell pellets were washed with ice-cold normal saline and then homogenized using 0.1 mm zirconia-silica beads in a Mini-BeadBeater (Biospec, Bartlesville, USA) at a speed of 4000 r.p.m. for 1 min, followed by cooling on ice for 1 min. This homogenization and cooling cycle was repeated five times. The samples were then centrifuged at 15 000 r.p.m. and the bacterial RNA in the supernatant was purified using RNeasy Mini Kits (Qiagen) and quantified using an ND-1000 spectrophotometer (NanoDrop Technologies, Wilmington, USA). RNA samples, which had a 260/280 ratio between 2.0 and 2.2 were reverse transcribed with the PrimeScript RT Reagent Kit (TaKaRa). Finally, qRT-PCR was performed with SYBR Premix Ex Taq II Kits (TaKaRa) and the Mastercycler ep realplex system (Eppendorf), with an initial incubation at 95 °C for 2 min, followed by 40 cycles of 15 s at 95 °C and 60 s at 60 °C. Each sample was analysed in triplicate.

For all samples, the internal control gene *recA* was used to normalize the expression of *esp, agg* and *cylA* [26]. The threshold cycle (Ct) numbers were determined by the detection system software and the data were analysed based on the 2^−△△Ct^ method.

### Statistical analysis

The prevalence of *E. faecalis* biofilms is shown as percentages and compared using the chi-square test. The virulence factors associated with biofilm formation were analysed with a multivariate logistic regression model. The logistic regression model was constructed by a backward selection approach based on the Wald statistic. *P-*values<0.05 were regarded as statistically significant. All data were analysed using the statistical software SPSS (version 14.0, Chicago, IL, USA).

## Results

### Biofilm formation by *E. faecalis* isolates from UTIs

OD_570_ microplate readings after CV staining ranged from 0.05 to 3.5 and were blanked with the non-inoculated reference well. Isolates with a biofilm phenotype were categorized as described previously [24], as strong (OD_570_≥1) or weak (OD_570_>0.5 and <1). The median OD_570_ values for controls was 0.55 for strain OG1RF (weak biofilm [27]) and 0.16 for strain ATCC29212 (negative biofilm).

The prevalence of *E. faecalis* biofilms from urinary tracts was 50.4 % (57/113) and the distribution characteristics of the biofilm phenotype were as follows: strong biofilms were 26.5 % (30/113) and weak biofilms were 23.9 % (27/113). Among the biofilm-forming isolates, more than half had a strong biofilm phenotype 52.6 % (30/57). There were 29 *E. faecalis* isolates from catheter-related UTIs in this study, and the prevalence of biofilm formation was similar between those catheter-related UTIs (14/29, 48.3 %) and catheter-unrelated UTIs (43/84, 51.2 %). The strong biofilm phenotype of *E. faecalis* was also similar between those catheter-related UTIs (7/29, 24.1 %) and catheter-unrelated UTIs (23/84, 27.4 %).

### Correlation between biofilm formation and virulence factors

The *E. faecalis* virulence factors were PCR amplified and analysed. As shown in Table 1, *esp-*positive isolates had more prevalent biofilm formation than *esp-*negative isolates (59.7 vs 30.6 %, *P=0.004*). Similarly, biofilm formation was more frequently detected in *asa1* (surface aggregating protein) and *cylA* (cytolysin A)-positive isolates than in negative isolates (respectively, 56.3 vs 17.6 %, *P=0.003* and 58.3 vs 27.6 %, *P=0.004*). Strong biofilm formation was more frequently detected in *agg-*positive isolates than in negative isolates (38.1 vs 19.7 %, *P=0.033*). However, *gelE-*negative isolates had greater biofilm formation than *gelE-*positive isolates (68.4 vs 41.3 %, *P=0.007*). This was particularly true for those strong biofilm-forming isolates (Table 1, see the footnote).

**Table 1.** Relationship between biofilm-forming capacity and virulence factors

**Virulence factors (no. of isolates tested)**	**No. (%) of isolates with biofilm phenotype**			***P****^a^*
	**Weak**	**Strong**	**All positive**	
*esp*+ (77)	21 (27.3)	25 (32.5)	46 (59.7)	*0.004*
*esp*- (36)	6 (16.7)	5 (13.9)	11 (30.6)	
*gelE*+ (75)	16 (21.3)	15 (20.0)	31 (41.3)	*0.007*
*gelE*- (38)	11 (28.9)	15 (39.5)*^b^*	26 (68.4)	
*asa1*+ (96)	25 (26.0)	29 (30.2)	54 (56.3)	*0.003*
*asa1*- (17)	2 (11.8)	1 (5.9)	3 (17.6)	
*cylA*+ (84)	25 (29.8)	24 (28.6)	49 (58.3)	*0.004*
*cylA*- (29)	2 (6.9)	6 (20.6)	8 (27.6)	
*hyl*+ (25)	5 (20.0)	7 (28.0)	12 (48.0)	0.782
*hyl*- (88)	22 (25.0)	23 (26.1)	45 (51.1)	
*agg*+ (42)	7 (16.7)	16 (38.1)*^c^*	23 (54.8)	0.480
*agg*- (71)	20 (28.2)	14 (19.7)	34 (47.9)	

*a,* Biofilm formation: + versus −; *b*, strong biofilm: *gelE-* versus *gelE*+, *P*=0.027; *c, s*trong or medium biofilm: *agg+* versus *agg-*, *P*=0.033.

### The association between biofilm formation and MLST

**Fig. 1.**
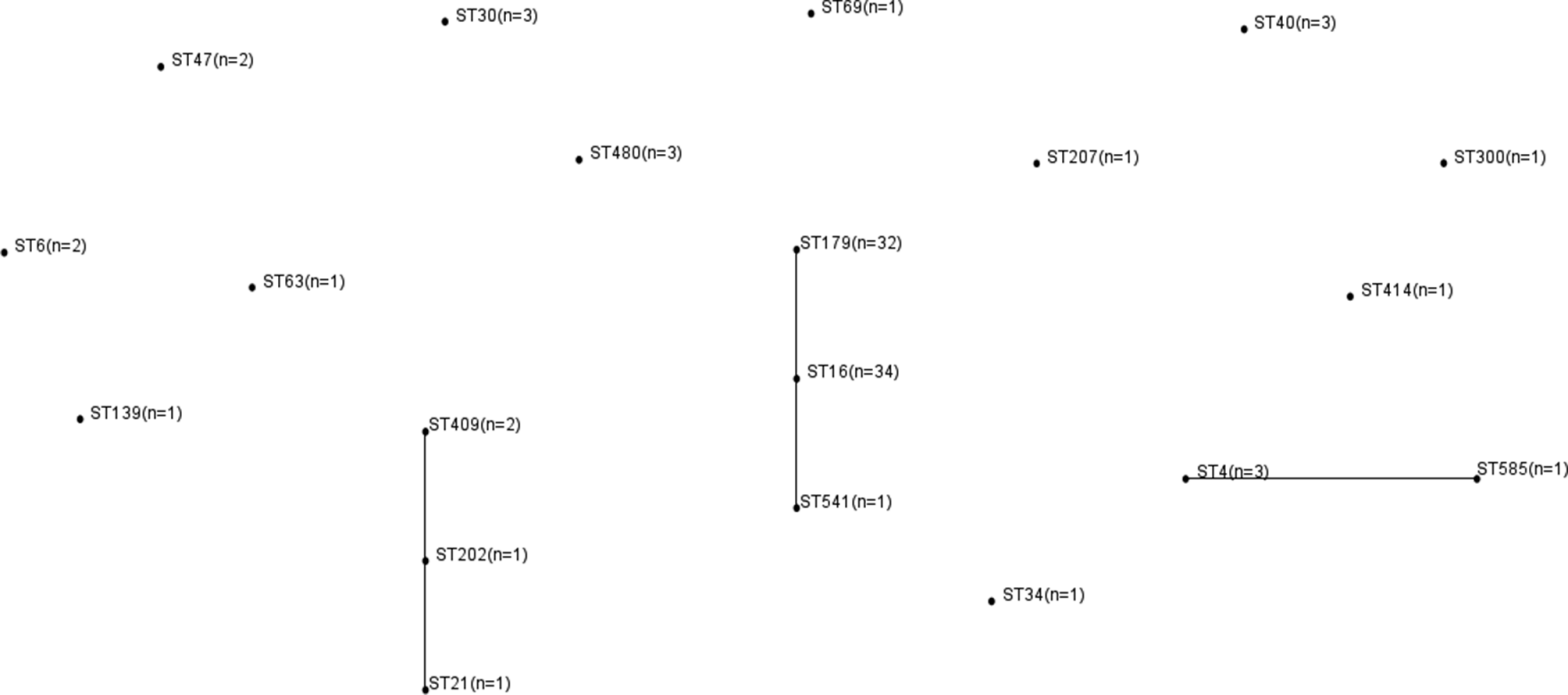
Distribution of MLST and clonal complex (CC) among 98 *E. faecalis* isolates. Connecting lines indicate CCs.

Of the 113 *E. faecalis* clinical isolates, 98 were identified successfully. The MLST of the remaining 15 *E. faecalis* isolates could not be determined due to the poor sequencing results despite multiple attempts. Overall, 23 different STs were identified and 21 of those were represented by four or fewer isolates. CC16 was predominant (Fig. 1). The biofilm characteristics and number of isolates assigned to each ST are reported in Table S4.

The main MLSTs of this study were ST16 and ST179, and these two STs accounted for 67.3 % (66/98) of the isolates. The relationship between *E. faecalis* biofilm formation and ST16 and ST179 was analysed with strong biofilm formation more frequently detected in ST16 isolates than in ST179 isolates (32.4 vs 6.3 %, *P=0.008*), Table 2. Total biofilm formation was also greater in ST16 isolates than in ST179 isolates (64.8 vs 31.3 %, *P=0.007*). *E. faecalis* virulence factors of ST16 and ST179 were analysed and *agg-*positive isolates were more common in ST16 isolates than in ST179 isolates (58.8 vs 15.6 %, *P<0.001*), Table 3. However, *gelE-*positive isolates were more prevalent in ST179 isolates than in ST16 isolates (87.5 vs 20.6 %, *P<0.001*).

**Table 2.** Biofilm-forming capacity for ST16 and ST179 isolates

**Biofilm phenotype**	**No. (%) with phenotype**		***P***
	**ST16(*n*=34)**	**ST179(*n*=32)**	
Weak	11 (32.4)	8 (25.0)	0.510
Strong	11 (32.4)	2 (6.3)	*0.008*
All positive	22 (64.8)	10 (31.3)	*0.007*

ST, sequence type.

**Table 3.** Virulence factor traits for ST16 and ST179

**STs (*n*)**	**No. (%) of isolates with virulence factors**					
	***esp+***	***gelE+***	***asa1+***	***cylA+***	***hyl+***	***agg+***
ST16 (*n*=34)	27 (79.4)	7 (20.6)	32 (94.1)	31 (91.2)	3 (8.8)	20 (58.8)*^a^*
ST179 (*n*=32)	27 (84.4)	28 (87.5)*^b^*	31 (96.9)	30 (93.8)	5 (15.6)	5 (15.6)

ST, sequence type; +, positive.

*a, agg+*: ST16 versus ST179, *P*<0.001; *b, gelE+*: ST179 versus ST16, *P*<0.001.

### Association of biofilm formation with antimicrobial susceptibility

The MICs of ampicillin, vancomycin and linezolid were tested by the broth macrodilution method, and the drug resistance was analysed based on the guidelines of CLSI for 2016. A total of 18 isolates were insensitive to linezolid (MIC>2 µl ml^−1^), four isolates were resistant to ampicillin, and none of the isolates were resistant to vancomycin or teicoplanin. Biofilm formation was not associated with antimicrobial sensitive or insensitive isolates for ciprofloxacin, tetracycline or high-level gentamicin, Table S5.

### Multivariate regression analysis of virulence factors associated with biofilm formation

In order to determine the independent contribution of each virulence factor to biofilm formation by *E. faecalis*, multiple logistic regression analysis was performed. Virulence factors, *esp*, *gelE*, *asa1*, *cylA, hyl (*hyaluronidase) and *agg,* were used as independent variables for weak biofilm formation, with strong biofilm formation used as a dependent variable in the multivariate model. The logistic regression model was constructed by a backward selection approach based on the Wald statistic. *P-*values<0.05 were regarded as statistically significant.

As Table 4 shows, for weak biofilm formation only *cylA* (OR, 7.143, 95 % CI, 1.539–33.140; *P=0.012*) was an independent risk factor. Other virulence factors, *esp*, *gelE*, *asa1* and *agg,* were not risk factors for *E. faecalis* weak biofilm formation. Virulence factor *agg* (OR, 4.471, 95 % CI, 1.593–12.552; *P=0.004*) was independently associated with strong biofilm formation. It is of interest that *gelE-*positive isolates (OR, 0.215, 95 % CI, 0.076–0.608; *P=0.004*) showed diminished strong biofilm formation.

**Table 4.** Multivariate regression analysis of virulence factors associated with *E. faecalis* biofilm formation

**Isolate biofilm phenotype**	**Factor**	**OR (95 % CI)**	***P***
*Weak biofilm formation*			
	*cylA*	7.143 (1.539–33.140)	*0.012*
*Strong biofilm formation*			
	*gelE*	0.215 (0.076–0.608)	*0.004*
	*agg*	4.471 (1.593–12.552)	*0.004*

OR, odds ratio; CI, confidence interval.

### High expressions of *agg* and *cylA* in *E. faecalis* biofilm positive isolates

**Fig. 2.**
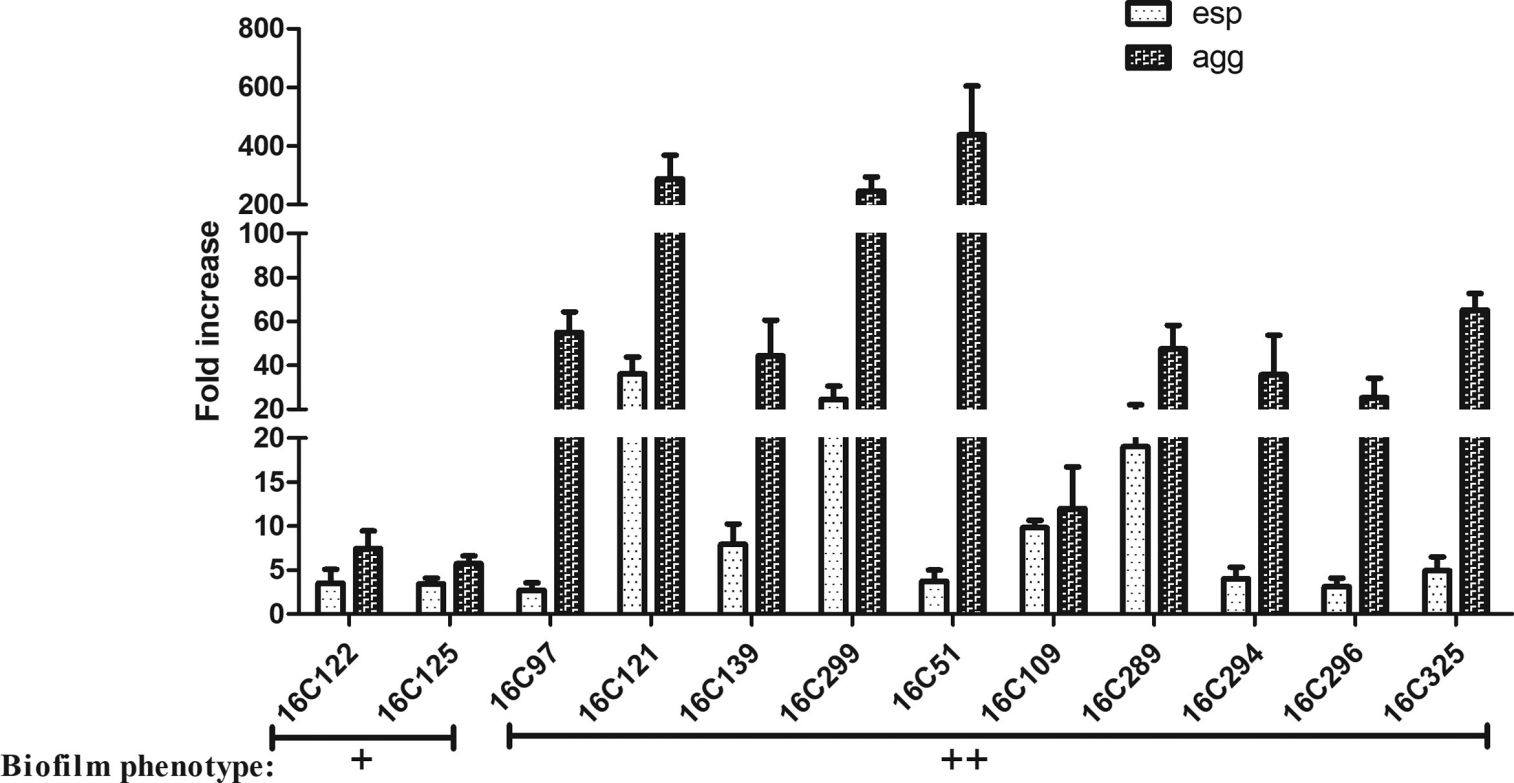
Expression levels of *esp* and *agg* in 12 clinical *E. faecalis* isolates. The expression levels of *esp* and *agg* were determined by qRT-PCR, with the clinical *E. faecalis* isolate 16C99 as the reference strain (expression=1, biofilm negative). Biofilm phenotype: **+**, weak; **++**, strong. In the strong biofilm isolates, *agg* was overexpressed (11.99- to 439.10-fold).

In order to verify the roles of *esp*, *agg* and *cylA* genes in biofilm formation, expression levels were determined by qRT-PCR. For the detection of *esp* and *agg*, 13 clinical *E. faecalis* isolates positive for *esp* and *agg* were selected. 16C99 was used as a negative biofilm reference strain (*esp* expression=1 and *agg* expression=1). As shown in Fig. 2, *agg* expression was markedly elevated (11.99- to 439.10-fold) in strong biofilm formation, compared to weak biofilm formation (16C125, 5.73-fold and 16C122, 7.45-fold). However, *esp* expression was mildly elevated (2.13- to 41.55- fold) in strong biofilm formation. These results suggest that *agg* plays a more important role than *esp* in strong biofilm formation by *E. faecalis* isolates from UTIs, which is consistent with previous findings.

**Fig. 3.**
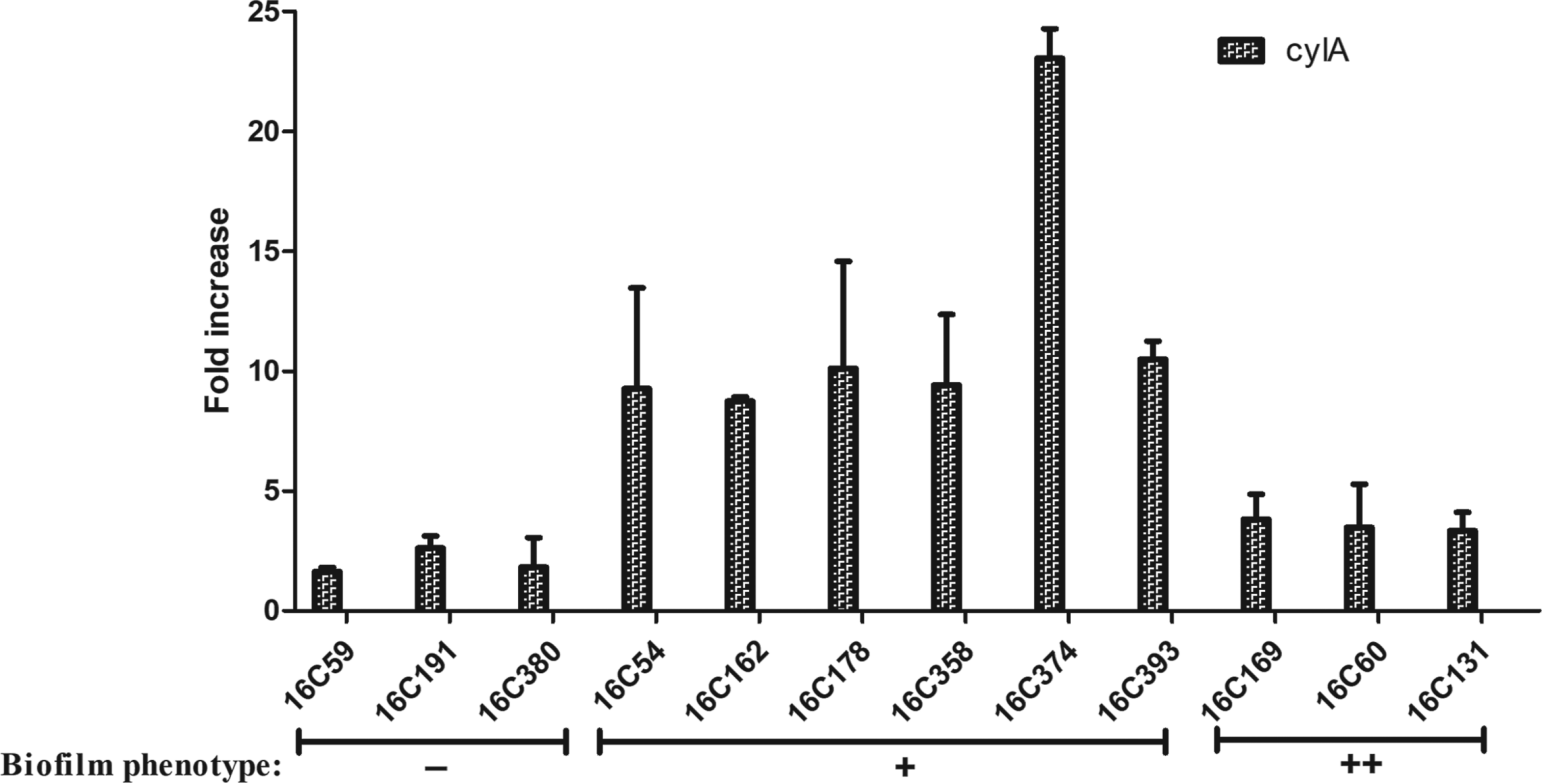
Expression levels of *cylA* in 12 clinical *E. faecalis* isolates. The expression levels of *cylA* were determined by qRT-PCR, with the clinical *E. faecalis* isolate 16C76 as the reference strain (expression=1, biofilm negative). Biofilm phenotype: −, negative; **+**, weak; **++**, strong. In the weak biofilm isolates, *cylA* was overexpressed (8.75- to 23.05-fold).

In total, 13 clinical *cylA* positive *E. faecalis* isolates were assessed for biofilm formation with the 16C76 strain used as a negative biofilm reference strain (*cylA* expression=1). As shown in Fig. 3, *cylA* gene expression was higher (8.75- to 23.05- fold) in those isolates that formed weak biofilms, compared with negative biofilm isolates (16C59, 1.64-fold; 16C191, 2.62-fold; 16C380, 1.83-fold), or strong biofilm isolates (16C169, 3.82-fold; 16C60, 3.49-fold; 16C131, 3.34-fold). These data demonstrate *cylA* to be associated with weak biofilm formation and not with strong biofilm formation by these *E. faecalis* UTI isolates.

## Discussion

*E. faecalis* has become a common UTI pathogen. The drug resistance of *E. faecalis* is less than that of *E. faecium* but the prevalence of *E. faecalis* biofilm formation is higher than that for *E. faecium* [28] even though differences in incidence vary from one study to another. For example, 100 % of urinary tract *E. faecalis* isolates in Japan formed biofilms, while in India the prevalence was less than 80 % [6, 7]. In this study 50.4 % formed biofilms, significantly lower than those studies. Differences may be due to differing STs. The prevalence of catheter-related UTIs in this study was 25.7 %, which was similar to previous research (30.4 %) [6]. This study found *E. faecalis* from catheter-related UTIs was not associated with increased biofilm formation, and this is consistent with a previous study [6].

In China, the role of different *E. faecalis* STs in biofilm formation and the relationship of these to MLST was previously unknown. This study found 23 different STs, with ST16 and ST179 predominant. This result is similar to a previous report from Malaysia that found ST6, ST16, ST28, ST179 and ST399 in *E. faecalis* isolates [29]. Herein biofilm formation by ST16 and ST179 was significantly different even though these two STs belong to the same CC group. ST16 isolates produced more biofilms with stronger biofilm formation than ST179 isolates. This difference may be due to the expression of more *agg* and less *gelE* by ST16 isolates than ST179 isolates. Previously, ST16 isolates have been associated with hospital-adapted isolates [22]. Bacterial biofilms are more resistant to adverse environmental conditions and are more resistant to antimicrobials and disinfectants than planktonic bacteria [30]. Hospital environments rather than community environments have more antimicrobials and disinfectants, thus biofilm formation among hospital-adapted isolates is more likely than community-acquired isolates. ST16 of *E. faecalis*, like ST27 of *Staphylococcus epidermidis*, may have arisen in nosocomial environments, where there is a higher prevalence of STs that form biofilms [10].

Virulence factors of *E. faecalis* that contribute to biofilm formation are controversial. Previous studies have associated *esp* with *E. faecalis* biofilm formation [12, 13] and *esp* was shown to enhance *E. faecalis* biofilm formation [31]. As mentioned above, a previous Japanese study found *E. faecalis* with *esp* to form biofilms at a significantly higher rate than those without the gene [6]. Another study from India found both medium and strong biofilm isolates to be *esp-*positive [7]. In contrast, a study of clinical and laboratory *E. faecalis* isolates found no significant relationship between *esp* and *E. faecalis* biofilm formation [16–18]. In this study no association of *esp* with either weak or strong biofilm formation was found. Clarification of the virulence factors involved in *E. faecalis* biofilm formation requires further investigation.

Previous studies have found *gelE*, which hydrolyses gelatin, collagen and haemoglobin, to be associated with the development of *E. faecalis* biofilms [14, 15, 32]. However, in a large collection of *E. faecalis* isolates no significant relationship was found between *gelE* and biofilm formation [17, 18]. This study found biofilm formation to be more common in *gelE-*negative isolates than in *gelE-*positive isolates. This was particularly true for those isolates with the capacity to form strong biofilms. It may be that the gene sequence types of *E. faecalis* isolates in this study differ from those previous studies, or that unknown factors contribute to *E. faecalis* strong biofilm formation.

In addition to *esp* and *gelE*, previous studies have reported that aggregating substance promotes *E. faecalis* biofilm formation [19]. It is a plasmid-encoded bacterial adhesin that mediates efficient contact between donor and recipient bacteria, facilitating plasmid exchange [33]. In addition to its adhesive function during bacterial conjugation, *agg* mediates adhesion of *E. faecalis* to a variety of eukaryotic cells *in vitro*. This study confirmed that *agg* plays an important role in strong biofilm formation, but has no effect on weak biofilm formation. The role of *agg* in *E. faecalis* biofilm formation is under reported and requires further investigation.

This study has shown that *E. faecalis* isolates from UTIs easily form biofilms. The MLSTs of *E. faecalis* in this study were diverse, with ST16 and ST179 predominant. ST16 isolates displayed a higher biofilm-forming capacity than ST179 isolates. Further, the *cylA* gene was associated with weak biofilm formation, while *agg* was associated with strong biofilm formation by *E. faecalis* isolates from UTIs.

## Supplementary Data

151 Supplementary File 1
