# Supplementary material for: Characterization of biofilm formation by Enterococcus faecalis isolates derived from urinary tract infections in China
Source: J Med Microbiol. 2017 Nov 17;67(1):60–7. doi: 10.1099/jmm.0.000647 (PMC5882073; doi:10.1099/jmm.0.000647)
Supplement: Supplementary File 1 [file jmm-67-60-s001.pdf]

Table S1. PCR primers used for detection of *E. faecalis* virulence factors.

Target	Primer	Primer sequence (5'-3')	Amplicon size (bp)	Ref.
esp	*esp*-F	AATTGATTCTTTAGCATCTGG	510	[20]
	esp-R	AGATTCATCTTTGATTCTTGG		
gelE	*gelE*-F	TATGACAATGCTTTTTGGGAT	213	[20]
	gelE-R	AGATGCACCCGAAATAATATA		
asa1	*asal*-F	GCACGCTATTACGAACTATGA	375	[20]
	asal-R	TAAGAAAGAACATCACCACGA		
cylA	*cylA*-F	ACTCGGGGATTGATAGGC	688	[20]
	cylA-R	GCTGCTAAAGCTGCGCTT		
hyl	*hyl*-F	GACTGACGTCCAAGTTTCCAA	276	[20]
	hyl-R	ACAGAAGAGCTGCAGGAAATG		
agg	*agg*-F	CCAGTAATCAGTCCAGAAACAACC	406	[21]
	agg-R	TAGCTTTTTTCATTCTTGTGTTTGTT		

Table S2. PCR primers used for *E. faecalis* MLST gene diversity determination.

Target	Primer	Primer sequence (5'-3')	Amplicon size (bp)	Ref.
gdh	*gdh*-F	GGCGCACTAAAAGATATGGT	530	[22]
	gdh-R	CCAAGATTGGGCAACTTCGTCCCA		
gyd	*gyd*-F	CAAACTGCTTAG CTCCAATGGC	395	[22]
	gyd-R	CATTTCGTTGTCATACCAAGC		
pstS	*pstS*-F	CGGAACAGGACTTTCGC	583	[22]
	pstS-R	ATTTACATCACGTTCTACTTGC		
gki	*gki*-F	GATTTTGTGGGAATTGGTATGG	438	[22]
	gki-R	ACCATTAAAGCAAAATGATCGC		
aroE	*aroE*-F	TGGAAAACTTTACGGAGACAGC	459	[22]
	aroE-R	GTCCTG TCCATTGTTCAAAAGC		
xpt	*xpt*-F	AAAATGATGGCCGTGTATTAGG,	456	[22]
	xpt-R	AACGTCACCGTTCCTTCACTTA		
yqiL	*yqiL*-F	CAGCTTAAGTCAAG TAAGTGCCG	436	[22]
	yqiL-R	GAATATCCCTTCTGCTTGTGCT		

Table S3. PCR primers used for detecting the expression levels of *esp*, *agg*, and *cylA* *E. faecalis* isolates by qRT-PCR.

Target	Primer	Primer sequence (5'-3')	Amplicon size (bp)	Source or Ref.
recA	*recA*-F	CGACTAATGTCTCAAGCACTAC	106	This study
	recA-R	CGAACATCACGCCAACTT		
esp	*Resp*-F	GCATCAGTATTAGTTGGT	196	This study
	Resp-R	TTCCTTGTAACACATCAC		
agg	*Ragg*-F	CGTTGATAAAGCAGTTGAT	130	This study
	Ragg-R	TTGTAGTTGGTCTACTTCTT		
cylA	*RcylA*-F	GGAGGATATGGTGACAAT	163	This study
	RcylA-R	TTACTTCTGGAGTTGCTAA		

Table S4. Biofilm formation according to ST of 98 *E. faecalis* isolates.

	No. (%) of isolates with biofilm phenotype		
**STs (n)**	**Weak**	**Strong**	**All positive**
**ST4 (3)**	2 (66.7)	0 (0.0)	3 (100.0)
**ST6 (2)**	1 (50.0)	0 (0.0)	1 (50.0)
**ST16 (34)**	11 (32.4)	7 (20.6)	22 (64.8)
**ST21 (1)**	0 (0.0)	0 (0.0)	0 (0.0)
**ST30 (3)**	0 (0.0)	0 (0.0)	0 (0.0)
**ST34 (1)**	0 (0.0)	0 (0.0)	0 (0.0)
**ST40 (3)**	0 (0.0)	2 (66.7)	2 (66.7)
**ST47 (2)**	0 (0.0)	1 (50.0)	2 (100.0)
**ST63 (1)**	1 (100.0)	0 (0.0)	1 (100.0)
**ST69 (1)**	0 (0.0)	1 (100.0)	1 (100.0)
**ST139 (1)**	0 (0.0)	0 (0.0)	1 (100.0)
**ST179 (32)**	8 (25.0)	0 (0.0)	10 (31.3)
**ST202 (1)**	0 (0.0)	0 (0.0)	0 (0.0)
**ST207 (1)**	1 (100.0)	0 (0.0)	1 (100.0)
**ST300 (1)**	0 (0.0)	0 (0.0)	0 (0.0)
**ST403 (1)**	0 (0.0)	0 (0.0)	0 (0.0)
**ST409 (2)**	0 (0.0)	0 (0.0)	1 (50.0)
**ST414 (1)**	0 (0.0)	0 (0.0)	0 (0.0)
**ST480 (3)**	0 (0.0)	2 (66.7)	3 (100.0)
**ST506 (1)**	0 (0.0)	0 (0.0)	0 (0.0)
**ST541 (1)**	0 (0.0)	0 (0.0)	1 (100.0)
**ST585 (1)**	0 (0.0)	1 (100.0)	1 (100.0)
**ST7480 (1)**	0 (0.0)	1 (100.0)	1 (100.0)
**Total (98)**	24(24.5)	15 (15.3)	51 (52.0)

STs: sequence type;

Table S5. Correlation between biofilm-forming capacity and antimicrobial sensitivity.

Antimicrobials	Sensitive or MIC (mg/L)	No. (%) of isolates with biofilm phenotype			P^a
		Weak	Strong	All positive	
**erythromycin**	Non-Sensitive (n=96)	26(27.1)	24(25.0)	50(52.1)	*0.407*
	Sensitive (n=17)	1(5.9)	6(35.3)	7(41.2)	
**ciprofloxacin**	Non-Sensitive (n=40)	10(25.0)	14(35.0)	24(60.0)	*0.133*
	Sensitive (n=73)	16(21.9)	17(23.3)	33(45.2)	
**tetracycline**	Non-Sensitive (n=102)	25(24.5)	29(28.4)	54(52.9)	*0.106*
	Sensitive (n=11)	2(18.2)	1(9.1)	3(27.3)	
**High-level gentamicin**	Non-Sensitive (n=63)	13(20.6)	19(30.2)	32(50.8)	*0.933*
	Sensitive (n=50)	14(28.0)	11(22.0)	25(50.0)	
**linezolid**	MIC >2 (n = 18)	5(27.8)	6(33.3)	11(61.1)	*0.323*
	MIC ≤ 2 (n= 95)	22(23.2)	24(25.4)	46(48.4)	

MIC: minimum inhibitory concentration; **High-level gentamicin:** >500mg/L; **Non-Sensitive**: including the resistant and medium isolates; **[a]:** biofilm phenotype-All positive**:** Non-Sensitive group vs Sensitive group;
